# Effects of Metformin on Ischemia/Reperfusion Injury: New Evidence and Mechanisms

**DOI:** 10.3390/ph16081121

**Published:** 2023-08-09

**Authors:** Estefanie Osorio-Llanes, Wendy Villamizar-Villamizar, María Clara Ospino Guerra, Luis Antonio Díaz-Ariza, Sara Camila Castiblanco-Arroyave, Luz Medrano, Daniela Mengual, Ricardo Belón, Jairo Castellar-López, Yanireth Sepúlveda, César Vásquez-Trincado, Aileen Y. Chang, Samir Bolívar, Evelyn Mendoza-Torres

**Affiliations:** 1 Advanced Biomedicine Research Group, Faculty of Health Sciences, Universidad Libre de Colombia, Seccional Barranquilla, Barranquilla 081001, Colombia; estefanie-osoriol@unilibre.edu.co (E.O.-L.); wendyp-villamizarv@unilibre.edu.co (W.V.-V.); mariac-ospinog@unilibre.edu.co (M.C.O.G.); luis-diazariza@unilibre.edu.co (L.A.D.-A.); sarac-castiblancoa@unilibre.edu.co (S.C.C.-A.); ricardoj-belonm@unilibre.edu.co (R.B.); jairoa-castellarl@unilibre.edu.co (J.C.-L.); 2 Allied Research Society S.A.S., Barranquilla 080001, Colombia; yanireth-sepulveda@unilibre.edu.co; 3 Global Disease Research Colombia, Barranquilla 080001, Colombia; 4 Healthcare Pharmacy and Pharmacology Research Group, Faculty of Chemistry and Pharmacy, Universidad del Atlántico, Barranquilla 081007, Colombia; lemedrano@mail.uniatlantico.edu.co (L.M.); dmengual@mail.uniatlantico.edu.co (D.M.); samirbolivar@mail.uniatlantico.edu.co (S.B.); 5 Escuela de Química y Farmacia, Facultad de Medicina, Universidad Andres Bello, Santiago 8370134, Chile; c.vasqueztrincado@uandresbello.edu; 6 Department of Medicine, Faculty of Medicine, Foggy Bottom Campus, George Washington University, Washington, DC 20052, USA; chang@email.gwu.edu

**Keywords:** metformin, ischemia-reperfusion, heart, cardiovascular diseases, cardioprotection

## Abstract

The search for new drugs with the potential to ensure therapeutic success in the treatment of cardiovascular diseases has become an essential pathway to follow for health organizations and committees around the world. In June 2021, the World Health Organization listed cardiovascular diseases as one of the main causes of death worldwide, representing 32% of them. The most common is coronary artery disease, which causes the death of cardiomyocytes, the cells responsible for cardiac contractility, through ischemia and subsequent reperfusion, which leads to heart failure in the medium and short term. Metformin is one of the most-used drugs for the control of diabetes, which has shown effects beyond the control of hyperglycemia. Some of these effects are mediated by the regulation of cellular energy metabolism, inhibiting apoptosis, reduction of cell death through regulation of autophagy and reduction of mitochondrial dysfunction with further reduction of oxidative stress. This suggests that metformin may attenuate left ventricular dysfunction induced by myocardial ischemia; preclinical and clinical trials have shown promising results, particularly in the setting of acute myocardial infarction. This is a review of the molecular and pharmacological mechanisms of the cardioprotective effects of metformin during myocardial ischemia-reperfusion injury.

## 1. Introduction

According to World Health Organization (WHO) global health estimates, cardiovascular diseases (CVDs) remain the leading cause of morbidity and mortality in the world, claiming 17.9 million lives each year [1]. CVDs are disorders related to the heart and circulatory system, and ischemic heart disease is the most frequent, causing an estimated annual mortality of nine million people. CVDs mainly affect men over 40 years and older adults, with a significant increase over 70 years of age; it is known that three-quarters of deaths belong to low- and middle-income countries [1]. In Latin America, CVD represents 2 million deaths and ischemic heart disease 73.6 deaths per 100,000 people per year [2]. During ischemia, due to the intimate relationship between oxygen supply and coronary blood flow, normal perfusion to the cardiomyocyte is interrupted, leading to the termination of aerobic metabolism, creatine phosphate depletion, and anaerobic glycolysis, causing mitochondrial dysfunction [3]. Mitochondria not only produce adenosine triphosphate (ATP), but also participate in pivotal metabolic processes and cell signaling. This is the reason why mitochondrial dysfunction results in low levels of ATP, energy stress, production of reactive oxygen species (ROS), oxidative stress and alteration in the ionic balance, mainly in calcium homeostasis [4].

The increase in mitochondrial and cytoplasmic calcium stimulates the transition of mitochondrial membrane permeability complemented with the opening of pores that allow the free flow of solutes < 15 kDa, which results in alteration of the colloid osmotic pressure leading to inflammation and activation of proteases and lipases. This results in necrosis of the cardiomyocyte [5]. It is important to remember that mitochondria occupy 35% of the volume of adult cardiomyocytes and provide about 90% of the ATP through oxidative phosphorylation. Although restoration of blood flow and reoxygenation of tissues after myocardial ischemia can partially restore cardiac function, it also induces additional damage of up to 50%, due to excessive production of ROS and calcium overload, which is related to rupture of the sarcolemma and amplification of the inflammatory response, which ends in a coordinated involution of the myocyte (apoptosis). All these events are part of the ischemia/reperfusion (I/R) injury [5].

Common risk factors, such as a diet high in saturated fat, smoking, hypertension, obesity, and hyperglycemia, lead to the development of endothelial dysfunction, which is closely related to the oxidative stress and vascular inflammation that finally lead to CVD, especially in elderly adults [1,6]. Inflammation also plays a critical role in the development of CVD, especially during I/R, where initially there is a decrease in oxygen levels and the energetic substrates that lead to deleterious processes such as oxidative stress, endoplasmic reticulum stress, acidification and finally inflammation, along with the release of cytokines, fibrotic factors and ROS and reactive nitrogen species, with the subsequent abrupt increase in oxygen supply in the affected area. Both mechanisms induce cell and tissue damage [7,8,9]. In fact, it has been proven that by using anti-inflammatory therapy with monoclonal antibodies directed towards the innate immunity pathway (specifically towards interleukin-1β) in patients with previous myocardial infarction and elevated inflammatory serum biomarkers (C-reactive protein), fatal and non-fatal cardiovascular outcomes are reduced, regardless of blood lipid levels. This suggests that inflammation in patients with CVD represents another therapeutic target [10].

Metformin, a common and affordable drug for hyperglycemia, has been shown to exert cardioprotective [11] and anti-inflammatory effects beyond glycemic control [12,13,14]. Metformin is a biguanide drug used as the first-line medication in the treatment of type 2 diabetes [12], and has been documented to inhibit complex I of the mitochondrial respiratory chain, leading to activation of the cellular energy sensor AMP-activated protein kinase (AMPK). AMPK is activated by increasing adenosine monophosphate (AMP), ATP and adenosine diphosphate (ADP)/ATP ratios. Activated AMPK restores energy homeostasis by turning on catabolic pathways generating ATP while switching off cellular processes consuming ATP [13]. AMPK plays an important role in numerous diseases, such as type 2 diabetes mellitus (DM), dyslipidemia, and non-alcoholic fatty liver, as well as control of infections such as tuberculosis, ischemia, inflammation, and the aging processes [15]. Evidence has identified the fact that metformin may have many other benefits, including protection against cardiac ischemia-reperfusion (I/R) injury, atherosclerosis, inflammation, oxidative stress, and reducing the risk of stroke and death caused by CVD [12,16,17,18,19,20].

The potential cardioprotective effect of metformin may extend beyond its AMPK-mediated effect [21]. Evidence has shown that the administration of metformin during reperfusion leads to the activation of several kinases of the Reperfusion Injury Salvage Kinase (RISK) pathway, in which phosphatidylinositol-3-kinase (PI3K) and Protein kinase B (Akt) are involved [22,23,24]. Similarly, it has been reported that activation of multiple RISK pathway protective kinases inhibits the opening of the mitochondrial permeability transition pore (mPTP) during reperfusion, thereby limiting the size of myocardial infarction (MI) [25]. Also, metformin inhibits cell death by suppressing inflammation during apoptosis, and protects against myocardial I/R injury by activating several pathways that end up controlling and inhibiting autophagy and regulating apoptosis. This cardioprotective effect is linked to Akt signaling pathway activation [25]. This review summarizes the recent preclinical and clinical literature on the mechanisms that explain the cardioprotective effects of metformin, with special emphasis on myocardial I/R injury [26].

## 2. Metformin

Metformin was synthesized for the first time in 1920. Metformin’s chemical composition is 1,1-dimethylbiguanide hydrochloride. This drug is composed of guanidine, a substance found in the *Galician officinalis* plant of French origin that was used at the end of the 19th century for the treatment of diabetic patients [27]. Some derivatives of guanidine other than metformin were synthesized and used in the treatment of diabetes; however, the toxicity of these compounds limited their use [28]. In further clinical trials, metformin demonstrated a moderate antidiabetic effect, good tolerance in most cases, and had the advantage of not presenting the adverse effects such as lactic acidosis, weight gain, or hypoglycemia that arose with phenformin and buformin [29]. However, despite being a relatively well-tolerated medication, careful administration in diabetic patients with chronic kidney disease is required. Specifically, metformin administration should be dose-adjusted in patients with estimated glomerular filtration rates (eGFRs) of less than 45 mL/min per 1.73 m^2^ and withheld in cases of eGFRs less than 30 mL/min per 1.73 m^2^ [30].

## 3. Classical Effects of Metformin

Metformin is an anti-hyperglycemic drug that has three defined mechanisms of action. The first mechanism consists of the inhibition of hepatic gluconeogenesis and glycogenolysis [30], which results in increased insulin sensitivity in muscle cells and adipose tissue, leading to improvement in peripheral glucose uptake and eventual consumption of glucose by muscles and peripheral tissues [31]. Lastly, it causes a delay in intestinal glucose absorption and inhibits gluconeogenesis in enterocytes [32]. There are numerous mechanistic pathways involved in the effects of metformin, including enzymatic inhibition of the mitochondrial respiratory chain, specifically the reduced nicotinamide adenine dinucleotide (NADH) dehydrogenase (complex I); glycerol-3-phosphate dehydrogenase; the activation of AMPK, which is currently known as a central regulator of intermediate metabolism [33]; the inactivation of protein kinase A, which reduces the effects of glucagon; the increase in cyclic adenosine monophosphate; and the improvement in the release of glucagon-like peptide-1, capable of suppressing glucagon secretion via pancreatic beta cell-stimulated insulin release [34].

The widely accepted mechanism of action of metformin is AMPK activation, in which metformin acts as an inhibitor of mitochondrial respiration at the level of complex I of the respiratory chain, which leads to activation of the AMPK pathway [25,35]. Once complex I is inhibited, the production of ATP is reduced and the intracellular concentration of ADP increases. Consequently, cellular levels of AMP increase, and finally AMPK is activated, which inhibits the expression of gluconeogenic enzymes (phosphoenolpyruvate carboxykinase and glucose-6-phosphatase). Likewise, it inhibits lipogenesis, which improves insulin sensitivity [36].

Another effect of metformin is the improvement in lipid metabolism by preventing and reversing steatosis and inflammation, in a mouse model of steatohepatitis [37,38]. Another review provided further support for the AMPK-Metformin relationship by establishing that the inhibitory phosphorylation of acetyl-CoA carboxylase by AMPK is essential for the control of lipid metabolism [39]. These studies also show that metformin inhibits the expression of gluconeogenic genes with or without the AMPK pathway, suppressing glucose production in the liver and lowering blood glucose levels by affecting the use of lactate by gluconeogenesis [37,38,39].

At cardiac level, metformin is known to modulate cardiomyocyte bioenergetics. Interestingly, there is a concentration-dependent effect on mitochondrial respiration. In a comprehensive study by Emelyanova et al. (2021), developed in human-induced pluripotent stem cells (hiPSC-CMs) [40], metformin at ≤2.5 mM, increased mitochondrial respiration, through AMPK activation. Conversely, metformin at concentrations of 5 mM inhibited oxygen consumption; however, this effect was not dependent on AMPK participation. In isolated mitochondria from human atrium, metformin decreased complex I-dependent mitochondrial respiration (at ≥1 mM concentration), consistent with the described binding sites for medicinal biguanides at the mitochondrial respiratory chain [41]. Consistently, with the respiratory chain inhibition, metformin increased the extracellular acidification rate [41]. This finding can be connected to the metabolic reprogramming towards glycolysis exerted by metformin, since it raised lactate levels, among others the glycolysis metabolites [41].

Consistent with the above results, adult cardiomyocytes isolated from metformin-treated (100 mg/kg/day) mice exhibited a decreased oxygen consumption and an elevated extracellular acidification rate [42]. Supporting these results, metformin-treated hearts exhibited a reduced NAD^+^/NADH ratio [42], which can be a consequence of mitochondrial complex I blockade. In summary, metformin effects on cardiac bioenergetics are dose-dependent. Low doses increased oxygen consumption, which can be explained via AMPK activation and the subsequent increase in mitochondrial biogenesis. In contrast, high metformin doses reduced mitochondrial respiration, due to inhibition of complex I activity, and consequently enhanced glycolytic flux and lactate production. These findings demonstrate that in addition to its effects on glucose and lipid metabolism, metformin would also significantly affect cardiomyocyte bioenergetics. This is especially important considering the pivotal role that the cardiomyocytes play in myocardial contractility.

## 4. Myocardial Ischemia/Reperfusion (I/R) Injury

By 2030, there will be 23.6 million deaths attributed to CVD [43]. In 2019, heart attacks and strokes caused 85% of CVD deaths and CVDs were responsible for 38% of the 17 million premature deaths (before the age of 70) caused by noncommunicable diseases [1]. By far the most common underlying cause of coronary artery disease (CAD), carotid artery disease, and peripheral arterial disease, is atherosclerosis, which leads to an MI, heart failure, stroke, claudication, and premature death [43].

CAD is a cardiovascular pathology characterized by total or partial obstruction of the coronary arteries, which are responsible for supplying oxygen to the heart tissue [40]. The pathophysiology of the CAD is inflammatory and includes a series of interrelated processes that involve lipid and endothelial alterations, platelet activation, and thrombosis and oxidative stress, which together generate myocardial ischemia, whose clinical manifestation is angina, infarction, sudden cardiac arrest, or death [44]. Since 1990, according to the latest WHO report, CAD leads global mortality, being responsible for 16% of all deaths in the world [45]. The American Heart Association reports that a heart attack occurs approximately every 40 s in the United States, with an overall incidence of CAD of 49% for men and 32% for women older than 40 years [46]. It is estimated that 11% of adults over 45 years of age and 17% of those over 65 years of age have CAD, which generates great costs for health systems. In 2010, CAD represented an expense of USD 162.2 billion, and it is predicted that it will increase to more than USD 177 billion by the year 2040 [47]. Treatment must be comprehensive, and includes lifestyle modification, drug therapy, and revascularization, with further consideration given to the processes that lead to myocardial stunning during reperfusion [48].

Myocardial I/R injury is a complex process that is associated with mitochondrial damage and apoptosis, which can lead to loss of myocardial cells, and therefore reduced cardiac function [49]. When the blood flow through one or more of the coronary arteries is reduced, myocardial ischemia occurs [50], and the restoration of coronary arterial blood flow (reperfusion) causes significant damage to the cardiomyocytes [51]. During decreased blood flow, cellular metabolism that was previously aerobic becomes anaerobic, releasing metabolites such as lactate, free radicals, and other substances that generate toxicity in the cell and produce marked decreases in phosphocreatine levels [50]. During reperfusion, cell death occurs by both necrosis and apoptosis. Necrosis occurs because of pH normalization that favors the opening of the mitochondrial membrane transition pores [51,52] and the immune response triggered by the recognition of a “damage-associated molecular pattern” through toll-like receptors [53]. On the other hand, apoptosis is favored by the decrease in, or absence of, aerobic cellular respiration [51,52], the increase in ROS [51,52,53,54] and the release of tumor necrosis factor alpha (TNF-α) by activated macrophages, which bind to the TNF receptor, which subsequently fuses with protein 3, generating an apoptotic cascade [51]. All these processes generate inflammation. The inflammatory response starts with an accumulation of leukocytes and intercellular adhesion molecules, such as Intercellular Adhesion Molecule 1, which appears on the surface of myocytes after interleukin 6 (IL-6)-mediated stimulation of leukocytes [50]. Furthermore, neutrophils and monocytes induced by reperfusion release toxic substances for endothelial and myocardial cells, including free radicals, proteases, elastases, and collagenases [55]. Damage to endothelial cells produces alterations in vasorelaxation by interfering with the antithrombotic mechanisms that take place in the endothelium. This is when the induction of an inflammatory response occurs and allows an increase in capillary permeability, favoring platelet adhesion and the infiltration of leukocytes into the myocardium [55,56,57]. These inflammatory processes are also accompanied by other phenomena such as platelet activation, mediated in part by the action of free radicals, which can also cause tissue damage, and others such as the regulation of vascular tone, with a vasodilatory effect that occurs due to the presence of nitric oxide (NO) [58].

As previously presented, myocardial injury due to ischemia-reperfusion begins with an alteration in the metabolism of the cardiomyocyte that ends with the cell death and the consequent inflammatory response, which in recent years has been shown to play a crucial role. For this reason, in recent years interest has increased in the identification of molecules that can prevent or reduce the metabolic impact that the myocardial I/R injury produces, and thus preserve the integrity of the cardiomyocyte and the cardiac function.

## 5. Cardioprotective Effects of Metformin on Myocardial I/R

**Figure 1:**
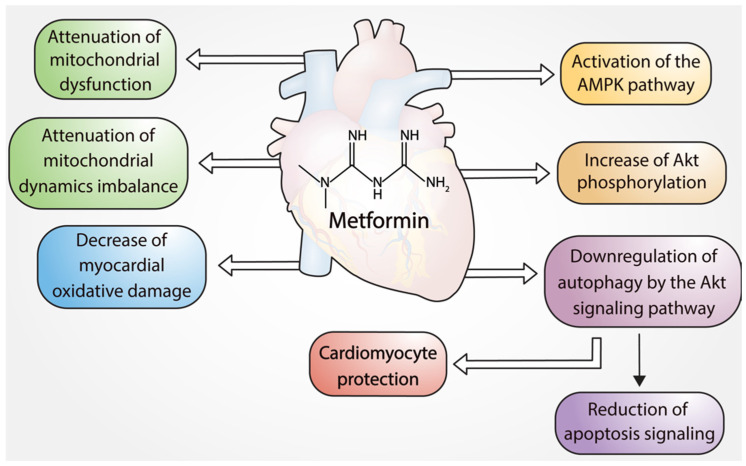
Cardioprotective effects of metformin.

In addition to being a popular antidiabetic, the literature suggests that there is an important cardioprotective effect in the use of metformin. Numerous preclinical studies have evaluated the effects of metformin on I/R-induced cell death and inflammation [59,60,61,62] (Figure 1).

### 5.1. Cardioprotective Effects of Metformin via Inhibition of Apoptosis

Palee et al. 2020 demonstrated in an experimental study that metformin treatment is cardioprotective in the setting of myocardial I/R injury, by attenuating mitochondrial dysfunction, mitochondrial dynamic imbalance, and apoptosis [62].

Some cardioprotective effects of metformin are mediated for AMPK. This kinase plays an important role in regulating myocardial energy metabolism, and reducing of I/R injury [62,63,64]. A recent experimental in vitro study performed by Zhang et al. showed that AMPK activation with metformin protected against apoptosis induced by cardiac I/R injury. In isolated rat hearts subjected to I/R, metformin reduced the size of the infarct and inhibited cardiac fibrosis through the reduction of proinflammatory cytokines (TNF-α, IL-6, IL-1β) and the suppression of the activation of the NLR family pyrin domain containing 3 (NLRP3) inflammasome [11]. Shi et al. showed in an experimental study that metformin reduced the oxidative stress injury induced by myocardial I/R. Metformin upregulated the phosphorylation of AMPK and decreased the NOX4 gene expression, leading to the decrease in myocardial oxidative damage and apoptosis, thereby alleviating reperfusion injury [64]. Metformin has also been reported to protect cardiomyocytes against H_2_O_2_-induced apoptosis through the AMPK/CCAAT-enhancer-binding protein (C/EBP) beta/miR-1a-3p/grpq4 (β/miR-1a-3p/GRPQ4) protein pathway. Through this pathway, Zhang et. al., determined that the expression of miR-1a-3p was significantly increased in neonatal rat ventricular cells which were exposed to H_2_O_2_ in vitro and in the hearts of mice that suffered I/R-induced injury. The miR-1a-3p follows protein GRP94, which results in the accumulation of unfolded or misfolded proteins, generating ER stress. C/EBP β directly induces the upregulation of miR-1a-3p, by binding to its promoter. Metformin appears to activate AMPK and at the same time significantly reduce the levels of C/EBP β and miR-1a-3p, compared to those of the control group in the study [65]. These in vitro and in vivo results confirm the positive effect of metformin for the treatment of cardiac I/R injury by attenuating cell apoptosis.

### 5.2. Cardioprotective Effects of Metformin on I/R Injury via Modulation of ROS

Metformin can additionally prevent I/R injury through other mechanisms such as modulation of ROS levels and oxidative damage during reperfusion. Metformin treatment reduced 4-HNE (4-hydroxynonenal) staining, an indicator of lipid peroxidation, after I/R injury [64]. This result is consistent with decreased NOX4 levels in metformin-treated hearts, which were dependent on AMPK activation. Interestingly, Asensio-Lopez et al., claimed that metformin protective effects over myocardial infarction, involved the participation of a mitochondrial NAPDH oxidase 4 (*mitoNox*), a mitochondria-localized NOX4 [66], which has been associated with increased ROS production during I/R and adverse myocardial remodeling (PMID: 25589557). Additionally, metformin has been shown to modulate ROS levels in I/R injury, through upregulation of antioxidant enzymes, such as MnSOD (manganese-containing superoxide dismutase) [16]. Similarly to other metformin effects, MnSOD upregulation was an AMPK-dependent process [16]. Thus, metformin has cardioprotective effects, involving oxidative stress management via AMPK activation, through reduction of pro-oxidant enzymes like (mito)NOX4, and upregulation of antioxidant enzymes such as MnSOD [64].

### 5.3. Cardioprotective Effects of Metformin on I/R Injury via Autophagy

Autophagy is an important physiological process in cells, which can degrade dysfunctional organelles and proteins. Autophagy plays a double role in myocardial I/R injury, and depends on the stimulus conditions. Moderate levels of autophagy can exert a protective effect against oxidative stress and the first minutes of hypoxia [64]; however, excessive activation of autophagy can cause cell death. Studies by Huang et al. and Wu et al. are an example of this double and opposite role of autophagy during I/R. Huang et. al. showed a destructive role of autophagy in myocardial I/R injury, and discovered that metformin inhibits apoptosis and inflammation during I/R via the restoration of autophagosome processing. Metformin induced the phosphorylation of Akt, which activates the mammalian target of rapamycin (mTOR), an inhibitor of autophagy. The density of autophagosomes in metformin-treated mice was much lower than in the control group, after I/R injury [65]. On the other hand, Wu et al. found that metformin improved cardiac function in infarcted rats previously treated with this drug, as evaluated by echocardiography, and promoted myocardial autophagy in in vivo models, thus revealing reductions in apoptosis and areas of infarction through the promotion of autophagy. Metformin promoted autophagy by increasing the protein expression of light chain 3 (LC3)-II, autophagy related (ATG) 5, ATG7 and Beclin1, and through the AMPK pathway during MI [67].

Fei et al. 2020 showed in their experimental study that metformin treatment in mice subjected to acute MI activated the autophagic flux in macrophages. Metformin decreased the expression of NLRP3 and p62 and increased the ratio of LC3II/LC3I. In addition, metformin treatment abrogated the effects of hydrogen peroxide and a combination of mitochondrial DNA (mtDNA) and ATP over the expression of NLRP3, and cleaved caspase-1 as well as intracellular ROS production in RAW264.7 macrophages. The suppression of NLRP3 by metformin was mainly attributed to the activation of the autophagy [68].

Metformin plays a dual role regarding autophagy in cardiomyocytes; it usually activates autophagy via AMPK or inhibits it in some models, via Akt/mTOR [25]. However, it is pertinent to consider that this difference may be due to the experimental conditions and models used. In fact, the role that autophagy plays in the context of cardioprotection is controversial; however, more studies are required to clarify whether autophagy is crucial in the cardioprotective mechanism of metformin.

Zhang et al. showed this dual action in an experimental study in murine models in which I/R myocardial damage was induced by transient occlusion of the left anterior descending artery. In this experimental model, the specimens were treated with metformin, showing a reduction in the ischemic area mediated by autophagosomes. The authors detected that the proteins associated with the membrane of the autophagosome LC3-II, BECLIN-1 and ATG5 were significantly increased in the group that received metformin; also shown was the increase in the expression of p-AMPK and the inhibition of p-mTOR, which represents an activation of autophagy through the regulation of this pathway with a partial reduction in myocardial damage and the subsequent cardioprotection [69].

### 5.4. Cardioprotective Effects of Metformin via Mitochondrial Function

Mitochondrial oxidative metabolism is the key source of cardiac energy, and mitochondrial dysfunction is considered the main myocardial mechanism that leads to a contractile failure after I/R injury. This organelle preserves its homeostasis and membrane potential through the mPTP, which is compromised by the irreversible myocardial damage that follows I/R injury, so there is a close relationship between both elements. Therapeutic strategies targeting I/R injury and also targeting mPTP inhibition have been developed to modulate energy homeostasis, mitochondrial function, and ROS production in cardiomyocytes. Specifically, it has been described that the translocator protein (TSPO) is the key component of mPTP that modulates these changes. Upregulation of TSPO expression was found to be associated with ROS accumulation and disruption of mitochondrial homeostasis. Myocardial TSPO expression was measured serially in correlation with the degree of mitochondrial homeostasis and cardiac function in a rat model exposed to myocardial I/R injury, using positron emission tomography imaging. The expression of TSPO was correlated with inflammatory changes in the infarcted area, and was associated with an upregulation of p-AMPK/AMPK; however, these effects were reversed with the use of metformin [70].

Aging has implications in the weakening of mitochondrial function, predisposing a greater cardiac injury during I/R. Stress on the cardiac endoplasmic reticulum (ER) increases with age, contributing to mitochondrial dysfunction. A study was conducted with mixed young (3 months) and old (2 years) mice; one group received metformin and sucrose water, while another group received sucrose alone, for 2 weeks. Cardioprotection was assessed using an isolated rat heart subjected to 25 min global ischemia and 60 min reperfusion, subsequently measuring the infarct size area. Factors highly involved in ER stress such as C/EBP homologous protein and cleaved activated transcription factor 6, were reduced in 24-month-old mice treated with metformin, compared to mice the same age that only received sucrose, which translates into a reduction in ER stress. In addition, metformin-treated rodents were found to have smaller infarct size after I/R [71].

**Figure 2:**
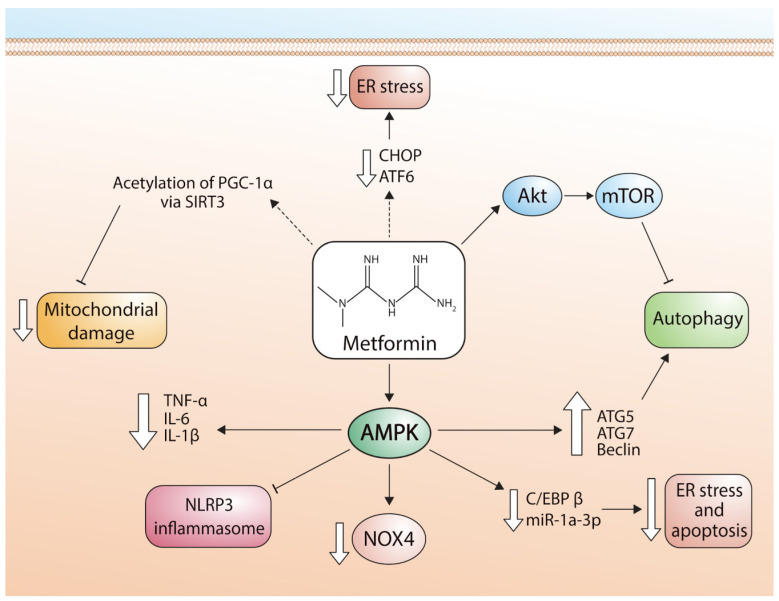
Pathways involved in the cardioprotective effects of metformin. ER stress: endoplasmic reticulum stress; CHOP: transcription factor CCAAT-enhancer-binding protein homologous protein; ATF6: activating transcription factor 6; Akt: alpha serine/threonine-protein kinase; mTOR: mammalian target of rapamycin; PGC-1α: peroxisome proliferator-activated receptor-gamma coactivator; SIRT3: Sirtuin-3; TNF-α: tumor necrosis factor alpha; IL-6: Interleukin 6; IL-1β: Interleukin 1 beta; NLRP3: NLR family pyrin domain containing 3; NOX4: NADPH oxidase 4; C/EBPβ: CCAAT/enhancer-binding protein beta; miR-1a-3p: microRNA-1 family; ATG5: autophagy related 5; ATG7: autophagy related 7.

In another experimental study, metformin treatment improved the mitochondrial respiratory function and mitochondrial membrane potential in male C57/BL6 mice subjected to myocardial infarction. The effects of metformin were evaluated during 8 weeks after MI, and this drug upregulated the expression of sirtuin (Sirt3) and the activity of peroxisome proliferator-activated receptor Γ coactivator 1 alpha (PGC-1α) in myocardial tissue of heart failure. Metformin decreased the acetylation of PGC-1α through the up-regulating of Sirt3 and thus mitigated the mitochondrial damage [72]. Metformin also attenuated I/R injury by decreasing mitochondrial dynamic imbalance and reducing cardiac mitochondrial dysfunction due to downregulation of ROS production, mitochondrial membrane depolarization, and swelling of the mitochondria [65]. Figure 2 describes the signaling pathways used by metformin to regulate mitochondrial function.

These preclinical findings support the ability of metformin to promote cardioprotection by reducing mitochondrial dysfunction, dynamic mitochondrial instability, and apoptosis, which decrease cardiac tissue injury. Therefore, it is plausible to postulate a potential clinical benefit of acute treatment with metformin in acute myocardial infarction.

Figure 2 shows the different pathways by which metformin exerts cardioprotective effects in cardiomyocytes via regulation of apoptosis, autophagy, and mitochondrial function (Figure 2).

### 5.5. Effects of Metformin on Cardiac Function after I/R Injury

The protective mechanism of metformin in cardioprotection is not fully understood. Nonetheless, several studies have investigated the effect of metformin during and after episodes of induced I/R. Su et al. (2022) developed an assay using murine models to determine the mechanism by which metformin attenuates cell damage caused by ischemia and subsequent reperfusion. They used rats subjected to occlusion of the left anterior descending coronary artery. The metformin group was treated with metformin twice a day for 30 days from the first day after the operation, while the control group was treated with saline solution for 30 days. The MI and fibrosis in the central ischemic area of the control group were more severe than those in the metformin group. The authors found that one month after the start of the intervention with metformin, the severity of the I/R event decreased, and ventricular remodeling was delayed. This study reported, for the first time in an in vivo molecular imaging assay, that metformin treatment improved glucose metabolic activity in ischemic myocardium and had a myocardial protective effect at the molecular level by delaying the development of ventricular remodeling triggered by I/R injuries [73].

Although more studies are needed to fully elucidate the action of metformin as a cardioprotective agent, current studies highlight how this drug protects cardiac function during myocardial injury. Jo et al. (2020) used murine heart models to demonstrate the effectiveness of metformin on left ventricular (LV) diastolic function using echocardiography in a rat myocardial I/R injury model. The model of myocardial I/R injury in rats involved tying off the left anterior descending coronary artery for 30 min, which resulted in MI-confirmed echocardiography [74].

Jo et al. (2020) confirmed the positive effect of metformin on the early stage of MI in rats through various assessments such as total weight change, relative heart weight, LV systolic and diastolic function using echocardiography on days 1, 3, and 7, and the degree of fibrosis. The LV diastolic dysfunction during MI is an important indicator of poor surgical outcomes and recurrence in human MI patients. The MI group exhibited decreased ejection fraction (EF) and fractional shortening values, reflecting LV systolic dysfunction, and also showed reduced E’ values (echocardiogram E wave) and increased E/E’ values; the E/e’ ratio refers to the relationship between the velocity of blood flow into the heart’s left ventricle during early diastole and the velocity of the mitral valve annulus during the same phase, reflecting LV diastolic dysfunction, which resembles human MI. The elevation of LV filling pressure is the key indicator of poor outcomes in human MI patients. This study also examined gene expression profiles, to provide a molecular basis for understanding the mechanism of action of metformin in this model [74]. The findings suggest that metformin can attenuate LV diastolic dysfunction induced by MI, and the molecular basis for this effect may involve alterations in immune/inflammation and cardiovascular system pathways, as well as fatty acid metabolism, mitochondrial biogenesis, and transforming growth factor-beta/bone morphogenetic protein and Janus kinase/signal transducers and activators of transcription signaling pathways. Overall, these results provide important insights into the potential clinical use of metformin in the treatment of MI, and highlight the need for further investigation of its effects on cardiovascular function [74].

Palee et al. (2020) investigated the efficacy of metformin in providing cardioprotection in a rat model of cardiac I/R injury. The results showed that metformin administration prior to cardiac I/R injury attenuated mitochondrial dysfunction, dynamic imbalance, and apoptosis, leading to a decrease in infarct size and improvement in cardiac function. The study also found that the optimal dose of metformin was 200 mg/kg, although doses of 100 and 400 mg/kg also provided some cardioprotective effects. These findings suggest that acute treatment with metformin may have clinical benefits for patients with MI. The reduction in excessive mitochondrial fission is important, as it is associated with cardiomyocyte apoptosis during cardiac I/R injury. The study also found that the reduction in phosphorylation of the cardiac gap junction protein Cx43 at serine 368 by metformin may have contributed to the higher mortality rate observed with a higher dose of metformin. Furthermore, the optimal dose and timing of metformin administration in the context of clinical relevance should be determined in future studies [75].

Eppinga et al. (2017) described in a randomized clinical trial the effects of metformin on the metabolic profiles of non-diabetic myocardial infarction patients and identified the prognostic metabolites predicting left ventricular ejection fraction (LVEF) and infarct size 4 months post-MI. Metformin treatment resulted in higher alanine levels and lower phospholipid content of large high-density lipoprotein (HDL) particles, as compared to controls. Higher triglyceride levels in HDL and several HDL subfractions measured 24 h post-MI were associated with favorable outcomes in terms of higher LVEF and smaller infarct size four months post-MI. The researchers also found that decreased HDL triglyceride levels measured 24 h post-MI predicted higher infarct size. The authors observed beneficial effects of higher levels in HDL measured 24 h post-MI on infarct size and LVEF [76].

The use of metformin in the management of cardiovascular disease has shown promising results, particularly in the setting of acute myocardial infarction. The cardioprotective effects of metformin may be attributed to its ability to modulate various metabolic pathways involved in energy metabolism, inflammation, and oxidative stress. Studies have demonstrated that the early administration of metformin following acute MI improves LV EF and reduces infarct size. Additionally, metformin has been shown to alter lipid profiles and increase the levels of cardioprotective HDL subfractions [76]. These findings suggest that metformin could be a valuable adjunct therapy in the management of acute MI and the prevention of recurrent cardiovascular events. However, further studies are required to establish the optimal dosing and duration of metformin therapy in different patient populations and to elucidate the underlying mechanisms of its cardioprotective effects. Table 1 describes some effects of metformin in preclinical studies, with experimental details.

## 6. Clinical Trials

The growing interest in the cardioprotective effects of metformin has allowed the development of various clinical trials with the aim of demonstrating this assumption in different scenarios. The greatest benefits have been attributed to diabetic patients. Table 2 shows some completed clinical trials in which the cardioprotective effects of metformin were evaluated:

Despite the fact that the results in clinical trials are controversial and the most promising results were observed in diabetic patients or those with cardiovascular risk factors, most authors agree that more studies should be carried out to evaluate the cardiovascular benefits of metformin in the long term.

## 7. Perspectives and Conclusions

Currently, the cardioprotective effects of metformin have been extensively studied. Metformin attenuates mitochondrial dysfunction and mitochondrial dynamic imbalance by protecting cardiomyocytes from I/R-induced apoptosis and inflammation through downregulation of autophagy, by activation of its canonical Akt pathway. On the other hand, metformin treatment has a beneficial effect on traditional risk factors for the development of atherosclerosis, and there is consistent evidence from animal studies that metformin therapy limits the infarct size in acute-MI remodeling, thus reducing the development of heart disease. Similarly, metformin, through the inhibition of the nuclear factor kappa-light-chain-enhancer of activated B cells pathway in macrophages, reduces the synthesis and release of proinflammatory molecules such as NO, proinflammatory cytokines such as IL-1β, IL-6 and TNF-α, and other molecules that play a pivotal role in the inflammatory process, such as prostaglandin E2. The evidence for the anti-inflammatory effects of metformin strengthens the arguments supporting its cardioprotective effects.

Furthermore, metformin may be considered for off-label therapeutic use beyond routine use, given the recent reduction in the contraindications of metformin in patients with heart disease by the FDA and the fact that it is a highly available and inexpensive drug. In addition, its therapeutic potential has been confirmed by its ability to reduce mortality and morbidity due to cardiovascular disease in patients with DM.

However, significant research questions remain. Since the results published in the literature are mainly of patients with DM, it is of vital importance to determine in depth the beneficial effects of metformin in patients without DM. The evidence in non-diabetics remains controversial, with multiple studies reporting little or no effect of metformin on measures of cardiovascular health in these patients. Therefore, obtaining further cardiovascular clinical results in patients treated with metformin without DM will depend on continuous follow-up and the development of new randomized clinical trials.

Our research supports that metformin can potentially serve in the prevention and treatment of CVD, which has been demonstrated by the existing literature on mechanistic studies in animal experiments and clinical trials in patients. However, more randomized controlled clinical trials are urgently needed; in particular, clinical trials with extended follow-up, to determine the benefits of prevention/delay of DM, are needed.

Inflammation has certainly been shown to be a major contributor to the development of cardiovascular disease. Therefore, a successful approach to inflammation in CVD will require new treatment paradigms. Scientific evidence strongly suggests that the anti-inflammatory effects of metformin should be further investigated, with a particular focus on its usefulness in non-diabetic cohorts. The metformin-induced inhibition of inflammation in preclinical models, as well as the decrease in injury caused by I/R, associated with mitochondrial damage and myocardial cell death by apoptosis, suggest that CVD patients with and without diabetes could benefit from metformin treatment.

## Figures and Tables

**Table 1:** In vitro and in vivo preclinical studies on the effects of metformin in I/R models.

Models	In Vitro	In Vivo	Dosage	Effects
I/R	H9C2 Cells	Sprague Dawley rats	In vitro: 0, 10, 20, 40 u 80 µmol/L In vivo: 250 mg/kg	Metformin reduced infarct size, increased STEAP4 expression and mitigation of myocardial apoptosis, and increased MMP when models underwent H/R or I/R lesions [59].
I/R	-	Sprague Dawley (SD) male rat	5 mg/kg	Metformin inhibited NOX4 expression through AMPK activation, resulting in decreased myocardial oxidative damage, apoptosis, and infarct size [60].
I/R	Neonatal Rat Ventricular Cardiomyocytes (NRVC)	-	0, 1, 0, 5, 1, 2, 5 o 10 mM	Metformin attenuated H_2_O_2_-induced cardiomyocyte injury via the AMPK/C/EBP β/miR-1a-3p/GRP94 pathway [64].
I/R	H9c2 Cells	C57BL/6 Mice	In vitro: 50 μM In vivo: 125 µg/kg	Metformin protected cardiomyocytes from I/R-induced apoptosis and inflammation through downregulation of autophagy mediated by Akt signaling pathway [65].
I/R	-	Sprague Dawley rats	400 mg/kg/day	Metformin decreased the size of the infarct in the heart by inducing autophagy through regulation of the mTOR/AMPK pathway [70].
I/R	Male Wistar rats	-	100, 200 y 400 mg/kg	Metformin reduced mitochondrial fission, apoptosis, arrhythmias, infarct size, and preserved left ventricular function, thus reducing the mortality rate [76].

**Table 2:** Main findings in clinical trials that evaluate the cardioprotective effects of Metformin.

Title and Authors	Treatment	Main Findings
Metformin use and cardiovascular events in patients with type 2 diabetes and chronic kidney disease. Charytan et al. (2019) [77].	591 individuals who used metformin at baseline and 3447 non-users	Cardiovascular mortality, cardiovascular events and the combined endpoint were lower in metformin users than in non-users.
Metformin Use and Clinical Outcomes Among Patients with Diabetes Mellitus With or Without Heart Failure or Kidney Dysfunction: Observations From the SAVOR-TIMI 53 Trial. Bergmark et al. (2019) [78].	Patients in SAVOR-TIMI 53 (saxagliptin and cardiovascular outcomes in patients with type 2 diabetes mellitus) were classified as ever versus never taking metformin.	Reduction in mortality after the use of metformin in association with other antidiabetic drugs.
Effect of metformin on cardiovascular risk factors in middle-aged Thai women with metabolic syndrome: A randomized placebo-controlled trial. Indhavivadhana et al. (2020) [79].	Double-blind and placebo-controlled study in 40 menopausal women with metabolic syndrome after taking metformin 1700 mg/day for 6 months.	Metformin improved some parameters of metabolic syndrome. Metformin improved body mass index, fasting blood glucose, high-sensitivity C-reactive protein and 10-year risk of coronary heart disease.
Effect of intensive lifestyle modification & metformin on cardiovascular risk in prediabetes: A pilot randomized control trial. Kulkarni et al. (2018) [80].	103 prediabetic patients were randomized into three arms: standard care (STD), intensive lifestyle modification (ILSM) or ILSM and metformin (ILSM + Met), and followed up for six months.	Reduction in weight and fasting blood sugar from baseline in all three arms. No difference in high-sensitivity C-reactive protein and carotid intima-media thickness in the two intervention arms, compared to standard care.
Effects of Metformin Therapy on Coronary Endothelial Dysfunction in Patients with Prediabetes With Stable Angina and Nonobstructive Coronary Artery Stenosis: The CODYCE Multicenter Prospective Study. Sardu et. al. (2019) [81].	258 propensity score-matched (PSM) patients with stable angina undergoing coronary angiography were classified into three groups: 86 with normoglycemia, 86 with prediabetes, and 86 with prediabetes treated with metformin.	Major cardiovascular events were lower in the group treated with metformin.
Effect of Metformin and Lifestyle Interventions on Mortality in the Diabetes Prevention Program and Diabetes Prevention Program Outcomes Study. Lee et al. (2021) [82].	3234 healthy patients with risk factors for type 2 diabetes mellitus were randomized and subjected to intensive lifestyle intervention, metformin, or placebo.	Metformin and lifestyle modification prevented diabetes. However, none of these strategies reduced cancer or cardiovascular mortality rates.
Two-year follow-up of 4 months metformin treatment vs. placebo in ST-elevation myocardial infarction: data from the GIPS-III RCT. Hartman et al. (2017) [83].	379 patients without diabetes undergoing primary percutaneous coronary intervention were randomized to a 4-month treatment with metformin or placebo.	Four months of metformin treatment in STEMI patients without diabetes did not exert favorable long-term effects.
Effects of Long-term Metformin and Lifestyle Interventions on Cardiovascular Events in the Diabetes Prevention Program and Its Outcome Study. Goldberg et al. (2022) [84].	3.234 people with impaired glucose tolerance were randomly assigned to receive metformin 850 mg twice daily, a strict diet, or a placebo, and were then monitored for three years. The authors also examined whether these interventions reduced the incidence of major cardiovascular events over a 21-year median follow-up.	Neither metformin nor lifestyle reduced major cardiovascular events.

## Data Availability

Not applicable.

## Abbreviations

ADP	Adenosine Diphosphate
AMP	Adenosine Monophosphate
AMPK	AMP-Activated Protein Kinase
ATG5	Autophagy Related 5
ATG7	Autophagy Related 7
ATP	Adenosine Triphosphate
CVD	Cardiovascular Diseases
DM	Diabetes Mellitus
eGFR	Estimated Glomerular Filtration Rates
HDL-cholesterol	High Density Lipoprotein—Cholesterol
HOMA-IR	Homeostatic Model Assessment of Insulin Resistance
hTERT	Telomerase Reverse Transcriptase—Human
I/R	Ischemia-Reperfusion
IL-1	Interleukin 6
IL-6	Interleukin-6
KLF2	Krueppel-Like Factor 2
LC3I	Light Chain 3 I
LC3-II	Light Chain 3 II
LDL-cholesterol	Low Density Lipoprotein Cholesterol
MMPs	Matrix Metalloproteinases
mtDNA	Mitochondrial DNA
mTOR	Mammalian Target Of Rapamycin
NADH	Reduced Nicotinamide Adenine Dinucleotide
NF-kB	Nuclear Factor-Κb
NLRP3	NLR Family Pyrin Domain Containing 3
NO	Nitric Oxide
NOX4	NADPH Oxidase 4
NRF-1	Nuclear Respiratory Factor 1
NRF-2	Nuclear Respiratory Factor 2
p62	P62 Protein
PDK4	Pyruvate Dehydrogenase Lipoamide Kinase Isozyme 4
PGC-1α	Peroxisome Proliferator-Activated Receptor Γ Co-Activator 1 A
PI3K	Phosphatidylinositol-3-Kinase
RISK	Reperfusion Injury Salvage Kinase
ROS	Reactive Oxygen Species
SIRT-1	Sirtuin 1
SIRT3	Sirtuin 3
SQSTM1	Sequestosome 1
STAT 3	Signal Transducer and Activator of Transcription 3
TNF-α	Tumor Necrosis Factor Alpha
WHO	World Health Organization
mPTP	Mitochondrial permeability transition pore
MI	Myocardial infarction
CAD	Coronary artery disease
IL-6	Interleukin 6
BECLIN-1	Beclin 1 Polyclonal Antibody
TSPO	Translocator Protein
ER	Endoplasmic reticulum
EF	Ejection fraction
LV	Left ventricular
LVEF	Left ventricular ejection fraction
C/EBP	CCAAT enhancer binding protein
β/miR-1a-3p/GRPQ4	beta/miR-1a-3p/grpq4
Akt	Protein kinase B
